# In Obese Patients With Type 2 Diabetes, Mast Cells in Omental Adipose Tissue Decrease the Surface Expression of CD45, CD117, CD203c, and FcϵRI

**DOI:** 10.3389/fendo.2022.818388

**Published:** 2022-03-15

**Authors:** David Lopez-Perez, Anaïs Redruello-Romero, Jesús Garcia-Rubio, Carlos Arana, Luis A. Garcia-Escudero, Francisco Tamayo, Javier Salmeron, Julio Galvez, Josefa Leon, Ángel Carazo

**Affiliations:** ^1^ Department of Pharmacology, Faculty of Pharmacy, University of Granada, Granada, Spain; ^2^ Research Unit, Instituto de Investigación Biosanitaria de Granada (ibs.GRANADA), Granada, Spain; ^3^ Surgery Unit, San Cecilio University Hospital, Granada, Spain; ^4^ Endocrinology and nutrition department, Virgen de la Luz University Hospital, Cuenca, Spain; ^5^ Department of Statistics and Operative Research, Faculty of Sciences, University of Valladolid, Valladolid, Spain; ^6^ Gastroenterology Unit, San Cecilio University Hospital, Granada, Spain; ^7^ Centro de Investigación Biomédica En Red para Enfermedades Hepáticas y Digestivas (CIBER-EHD), Center for Biomedical Research, University of Granada, Granada, Spain; ^8^ Clinical Management Unit of Digestive Disease, San Cecilio University Hospital, Granada, Spain

**Keywords:** mast cell, adipose tissue, obesity, type 2 diabetes, flow cytometry, bariatric surgery

## Abstract

The paradigm of mast cells in type 2 diabetes is changing. Although they were first considered deleterious inflammatory cells, now they seem to be important players driving adipose tissue homeostasis. Here we have employed a flow cytometry-based approach for measuring the surface expression of 4 proteins (CD45, CD117, CD203c, and FcϵRI) on mast cells of omental (o-WAT) and subcutaneous white adipose tissue (s-WAT) in a cohort of 96 patients with morbid obesity. The cohort was split into three groups: non-T2D, pre-T2D, and T2D. Noteworthy, patients with T2D have a mild condition (HbA1c <7%). In o-WAT, mast cells of patients with T2D have a decrease in the surface expression of CD45 (p=0.0013), CD117 (p=0.0066), CD203c (p=0.0025), and FcϵRI (p=0.043). Besides, in s-WAT, the decrease was seen only in CD117 (p=0.046). These results indicate that T2D affects more to mast cells in o-WAT than in s-WAT. The decrease in these four proteins has serious effects on mast cell function. CD117 is critical for mast cell survival, while CD45 and FcϵRI are important for mast cell activation. Additionally, CD203c is only present on the cell surface after granule release. Taking together these observations, we suggest that mast cells in o-WAT of patients with T2D have a decreased survival, activation capacity, and secretory function.

## Introduction

Type 2 diabetes (T2D) accounts for 90% of all diabetes cases. This condition is growing its incidence because of poor dietary habits and a sedentary lifestyle and is the ninth death cause worldwide (1). Moreover, T2D will affect 693 million people in 2045 (2). Uncontrolled T2D can lead to severe chronic conditions, including micro and macrovascular diseases (3, 4). Thus, causing enormous pressure on healthcare systems.

Adipose tissue stores the positive caloric imbalance mainly in the form of neutral lipids. However, if this is sustained over time, adipose tissue has to expand to deal with the demands (5). Interestingly, different depot locations of adipose tissue have different expression and secretory profiles (6). Omental white adipose tissue (o-WAT) has a reduced adipogenic capacity compared to subcutaneous white adipose tissue (s-WAT). Consequently, when adipose tissue expands, in o-WAT the amount of free fatty acids is higher than in s-WAT. Besides, this accumulation of free fatty acids causes lipotoxicity and inflammation (7, 8). Thus, o-WAT expansion is a risk factor for cardiometabolic diseases and T2D (9).

Classically, adipocytes and macrophages were considered the main cell types involved in adipose tissue expansion. Nevertheless, recent works from different authors suggest that mast cells are also pivotal in this process. Adipocytes are the cells in charge of energy storage. They uptake carbohydrates (mainly mono- and disaccharides) and lipids and store them in the form of triacylglycerides (5). To cope with the positive caloric imbalance and increase the storage capacity of adipocytes, adipose tissue must expand. Such can be achieved by adipocyte hypertrophy or hyperplasia (10, 11). Although both pathways can coexist, generally one predominates over the other. The choice between these pathways depends on the tissue microenvironment. Nevertheless, these pathways have different metabolic consequences. During adipocyte hyperplasia, the number of adipocytes, insulin sensitivity, and adiponectin secretion increase. Besides, the production of pro-inflammatory cytokines decreases. In this process, angiogenesis is crucial to avoid hypoxic conditions. Inversely, adipocyte hypertrophy increases the size of adipocytes and the secretion of pro-inflammatory cytokines. Furthermore, insulin sensitivity and adiponectin secretion decrease. In adipocyte hypertrophy angiogenesis is impaired, leading to adipocyte necrotic death by hypoxia. This triggers more tissue inflammation and the release of free fatty acids (10, 11). In short, adipose tissue expansion can be performed in a metabolically healthy (adipocyte hyperplasia with efficient angiogenesis) or an unhealthy way (adipocyte hypertrophy with impaired angiogenesis).

Adipose tissue-resident macrophages clean senescent adipocytes and lipoproteins. When they ingest enough lipids, they form intracellular droplets and differentiate into foam cells (12). These foam cells can interchange lipids with adipocytes and prevent the accumulation of toxic free fatty acids in the extracellular space (12, 13). Moreover, under physiological conditions, foam cells have an M2 phenotype that contributes to adipose tissue homeostasis (13). When adipocytes become apoptotic, foam cells phagocyte them to prevent the release of free fatty acids and thus restoring the homeostasis of the microenvironment (12). Nevertheless, when adipose tissue expands too much *via* adipocyte hypertrophy, the insufficient vascularization causes hypoxic death of adipocytes. Notably, the hypoxic death of adipocytes occurs *via* necrosis instead of apoptosis. This releases pro-inflammatory mediators that recruit peripheral monocytes and polarize them to M1 macrophages (10, 11). Additionally, in this context, foam cells also switch to an M1 phenotype (13). When this adipocyte hypertrophy is maintained over time, the hypoxic areas become greater (10, 11). Besides, when foam cells reach their storage capacity they undergo necrosis and release a vast amount of proinflammatory cytokines and free fatty acids (12). As a result, dead adipocytes are not optimally cleaned and free fatty acids accumulate in the extracellular space driving tissue malfunction (10–13). In o-WAT, free fatty acids reach the portal system and liver, causing hepatic toxicity and sometimes fatty liver disease and hepatic steatosis (8, 14, 15).

As mentioned before, while adipocyte hyperplasia contributes to insulin sensitivity and homeostasis, adipocyte hypertrophy promotes insulin resistance and tissue deterioration. One important checkpoint to determine which pathway will be employed is the angiogenic capacity of the tissue (10, 11). Noteworthy, angiogenesis is impaired in adipose tissue of patients with T2D (16). Although angiogenesis is a multicellular process, mast cells are crucial players. They release proteases to generate the physical space for the vessel, pro-angiogenic factors (VEGF, bFGF, TGF-beta), histamine to increase vascular permeability, and heparin (17).

Apart from their role in angiogenesis, mast cells have a plethora of receptors to interact with their microenvironment (18, 19). To do so, mast cells store intracellularly a vast amount of granules with different cargo (19, 20). These granules contain different membrane-bound proteoglycans that denote the internal cargo (21). Therefore, the activation of different receptors on mast cells can promote the release of different granules or vesicles. This differential release can modulate the microenvironment directly or by interactions with other cell types (19).

The paradigm of mast cells is changing. They were first identified as inflammatory cells because of their role in allergy. Additionally, since adipose tissue inflammation is a hallmark of T2D, it was thought that they play a deleterious role in adipose tissue (18). However, recently, it has been observed that mast cells have an important physiologic role. Such includes the release of growth factors and other bioactive molecules in response to signals from the microenvironment (19).

In adipose tissue, mast cells promote angiogenesis, lipid uptake by macrophages, and foam cell formation (17). Besides, they respond to high glucose levels secreting 15-deoxy-delta prostaglandin J2. This molecule binds PPARγ in pre-adipocytes triggering their differentiation to adipocytes (22, 23). In a nutshell, in adipose tissue, mast cells contribute to normoxia, free fatty acid clearance, and adipose tissue expansion *via* hyperplasia.

T2D causes alterations in the adipose tissue micro-environment. This includes the recruitment of proinflammatory leukocytes from peripheral blood and also changes in the metabolomic profile (8, 24). These changes affect resident cells including mast cells. Although some experiments have been performed in mice, contradictory results have been obtained (25, 26). Additionally, little is known about mast cells in T2D in humans. Nevertheless, recently, it was observed that the number of mast cells decreases in patients with T2D (27), but there is scarce information about phenotypic changes in mast cells.

## Materials and Methods

### Biochemical Parameters

For each patient, two blood tests were analyzed. In the first one, 6-9 months before the surgery, only fasting plasma glucose was analyzed. In the second one, just before the surgery, a complete analysis was performed. Blood tests were conducted in San Cecilio University Hospital by the clinical analysis laboratory within 24 hours. All tests were performed following approved protocols.

### Cohort

For this study, 96 patients were recruited in the San Cecilio University Hospital. All patients had morbid obesity and underwent bariatric surgery (gastric bypass or gastric sleeve). Patients were stratified into three groups (non-T2D, pre-T2D, and T2D) following the criteria of the American Diabetes Association (28). Patients were excluded from the cohort if they present at least one of the following disorders: type I diabetes, drug-induced diabetes, genetic diabetes syndromes, diseases of the exocrine pancreas, and autoimmune diseases. All patients provided their written informed consent.

### Sample Processing

Each patient gave two biopsies of adipose tissue from laparoscopic bariatric surgery at San Cecilio University Hospital (Granada, Spain). One biopsy was taken from the greater omentum, close to the stomach (o-WAT, omental white adipose tissue). The second biopsy was obtained near the surgical incision (s-WAT, subcutaneous white adipose tissue). After their extraction, both biopsies were conserved in ice and separated plastic jars with PBS. Later, visible blood vessels were eliminated from the samples. After, 2-2.5 g of each sample was weighed and cut into tiny portions. Subsequently, cut biopsies were digested in 10 ml of RPMI 1640 medium supplemented with 2 mg/ml type I collagenase (Sigma) and 5 mM CaCl2, for 2h, at 37°C. Then, each sample was washed with 35ml of PBS and filtered through a 1 mm sieve. After, samples were centrifuged at 900 x g for 10 minutes and spilled through a 100 μm filter. Later, the samples were centrifuged again at 900 x g for 10 minutes, and the pellet was kept since it contains the stromal vascular fraction. Finally, the pellet was resuspended in 500 μl of antibody staining buffer (PBS, 2% fetal bovine serum, 0.09% albumin, and 0.05% sodium azide) and mixed with an internal standard (BD Truecount Absolute Counting Tubes) following manufacturer instructions. The internal standard contains a suspension of autofluorescent beads with excitation and emission occurring through a wide spectrum of wavelengths. Since the internal control has size/complexity values far from any other cytometric population, it cannot be misled with cells.

### Antibody Staining and Flow Cytometry

The resuspended stromal vascular fraction was stained with 2 μl of fluorophore-conjugated antibodies or controls in Eppendorf tubes for 20 minutes at room temperature. Later, samples were fixed, and erythrocytes were lysed adding 1 ml of BD FACS Lysing Solution for 30 minutes. After that, samples were centrifuged at 3500 x g for 10 minutes, and pellets were resuspended in 500 μl of PBS. Then, samples were stored until the next day at 4°C. A FACS ARIA III equipment was employed to perform the flow cytometry, and data were acquired on a logarithmic scale. The fluorescence of the internal standard was used to normalize the signal obtained from the fluorophore-conjugated antibodies (**Supplementary Figures 1–4**).

The fluorophore-conjugated antibodies employed were: anti-CD117 APC (clone YB5.B8, BD), anti-CD45 PE-CF594 (clone HI30, BD), anti-FcϵRI PE-Cy7 (clone AER-37, BioLegend), and CD203c BV421 (clone NP4D6, BioLegend). The voltages employed were APC (560V), PE-CF594 (430V), PE-Cy7 (530V), and BV421 (400V). Isotype controls and compensation beads were purchased from BD Biosciences. Mast Cells were identified as CD45^+^ CD117^+^ cells.

### RNA Purification and RT-qPCR

Total RNA was purified for each adipose tissue biopsy using the RNeasy Mini Kit (Qiagen). Five hundred nanograms of RNA were retrotranscribed to cDNA using the iScript™ cDNA Synthesis Kit (BioRad) according to the manufacturer’s instructions. The quantification of mRNA concentration for each gene was performed in a fraction of cDNA volume by Real-time PCR (CFX96 Real-Time System, BioRad) using the SsoFast EvaGreen Supermix (BioRad).

The primers (**Supplementary Table 1**) were tested previously to evaluate their specificity and sensitivity. Unspecific amplification was not detected in the test. To prevent amplification from eventual genomic DNA contamination, primers were designed to hybridize in different exons. Furthermore, when different transcript variants are described, primers were designed using the common sequence among them.

The annealing temperature was 65 °C. Each determination was carried out in duplicate, and the mathematical relation between the threshold cycle (Ct) level and the initial DNA quantity was evaluated by a standard curve. Finally, the results were normalized using the expression level of two housekeeping genes: PPIA (Peptidylprolyl isomerase A) and RPS13 (Ribosomal Protein S13).

### Statistical Analysis

Kolmogorov-Smirnov test was used to test the normal distribution of data, and the Levene test was employed to check the homoscedasticity of the groups. To evaluate the differences between non-T2D, pre-T2D, and T2D groups, the one-way ANOVA test was used followed by a Tukey HSD. Student’s t-test for paired samples was employed to analyze the differences between o-WAT and s-WAT, in non-T2D, pre-T2D, and T2D groups independently. Besides, we studied the internal structure of the data using the principal component analysis (PCA), multiple regression analysis, linear discriminant analysis, and random forest analysis. In the linear discriminant analysis and random forest analysis, we did not include the HbA1c variable because it is what defines the groups. P-values below 0.05 were considered significant. All tests were conducted with R software.

## Results

### Cohort Baseline Characteristics

**Table 1** shows the baseline characteristics of each group. Of note, females account for 2/3 of the cohort. Such may be because of social constraints in our geographic zone. Besides, patients in the T2D group are older than patients in pre-T2D and non-T2D. Such occurs because age is an important risk factor in the development of T2D.

**Table 1:** Cohort baseline characteristics.

	Non-T2D	Pre-T2D	T2D
**Number of patients**	34	32	30
**Age (years)**	42,8 ± 9.7	47.9 ± 13.2	52.1 ± 8.3
**Male/Female)**	12/22	11/21	8/22
**Hypertension (Yes/No)**	18/16	20/12	23/7
**Body Mass Index (kg/m^2^)**	44.9 ± 7.1	45.3 ± 6.6	43.9 ± 6.3
**Waist/Hip Index**	0.88 ± 0.10	0.92 ± 0.07	0.94 ± 0.07
**Insulin (units/ml)**	4.4 ± 3.3	7.6 ± 7.2	10.1 ± 6.9
**Glucose (mg/dl)**	86.9 ± 9.4	98.6 ± 15.3	156.1 ± 46.6
**HbA1c (%)**	5.2 ± 0.3	5.7 ± 0.5	6.8 ± 0.7
**HOMA-IR**	1.05 ± 0.53	1.97 ± 1.68	3.91 ± 2.44
**Triglycerides (mg/dl)**	138.3 ± 55.2	161.4 ± 69.4	152.0 ± 40.1
**Cholesterol (mg/dl)**	161.8 ± 39.5	152.5 ± 28.1	149.6 ± 43.8
**LDL (mg/dl)**	93.8 ± 34.0	85.1 ± 24.6	87.5 ± 43.6
**HDL (mg/dl)**	41.3 ± 11.4	37.1 ± 10.4	37.6 ± 12.0

Noteworthy, patients in the T2D group have a low HbA1c mean value, consistent with incipient or newly diagnosed T2D. In T2D patient management, the goal in most patients is to have HbA1c values below 7% since it considerably lowers the probability of having cardiovascular disease (29). Therefore, the patients with T2D in our cohort have a mild condition.

### Flow Cytometry of Mast Cells in o-WAT and s-WAT

**Figure 1:**
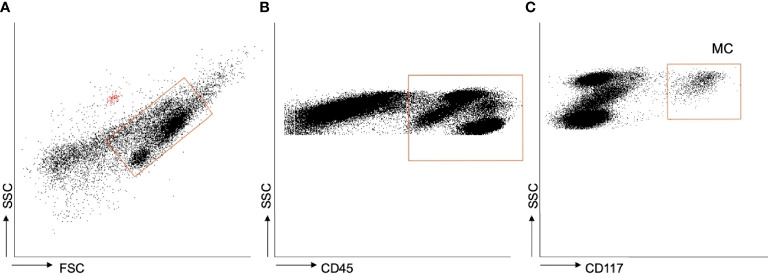
Flow cytometry. **(A–C)** Gating strategy employed to identify mast cells in adipose tissue. The red dots in plot A are the autofluorescent beads employed as internal standard.

**Figure 1** shows the gating strategy employed during flow cytometry. Mast cells were identified in the whole cohort as CD45^+^ CD117^+^ cells. **Supplementary Figures 1–4** show the fluorescence of each fluorochrome and the autofluorescent beads.

### The Surface Expression of CD45 on Mast Cells Decrease in Patients With Pre-T2D and T2D in o-WAT

**Figure 2:**
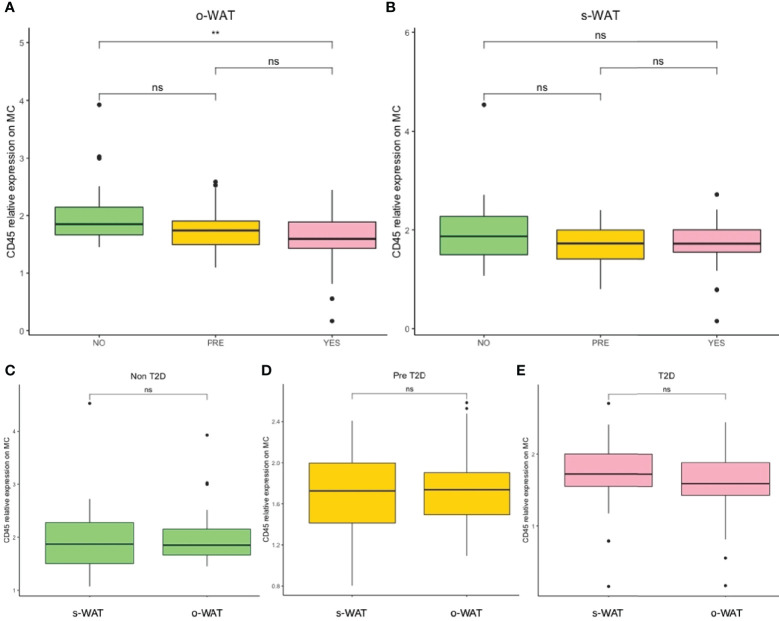
Relative surface expression of CD45 on mast cells. **(A)** Comparison in o-WAT between the three groups. **(B)** Comparison in s-WAT between the three groups. **(C)** Comparison between o-WAT and s-WAT in the non-T2D group. **(D)** Comparison between o-WAT and s-WAT in the pre-T2D group. **(E)** Comparison between o-WAT and s-WAT in the T2D group. MC (mast cells), s-WAT (subcutaneous white adipose tissue), o-WAT (omental white adipose tissue), NO (non-type 2 diabetes group), PRE (pre type 2 diabetes group), YES (type 2 diabetes group), T2D (type 2 diabetes), n=96. ** (0.01 > p value > 0.001). “ns” means not significant.

**Figure 2** shows the relative amount of CD45 on the surface of mast cells in the three groups (n=96). In the case of o-WAT (**Figure 2A**), CD45 decreases with T2D. However, differences were only statistically significant between non-T2D and T2D (p=0.0013). These results indicate that subtle changes in glycemic control have a huge effect on the surface expression of CD45 on mast cells in o-WAT. In s-WAT (**Figure 2B**), CD45 also decreases with T2D, although the differences are not statistically significant in any case.

When comparing o-WAT and s-WAT in the same patient in the three groups, there are no significant differences between the amount of CD45 on the surface of mast cells (**Figures 2C–E**). Nevertheless, in the case of the T2D group, differences are almost significant (p=0.072).

### The Surface Expression of CD117 on Mast Cells Decrease in Patients With Pre-T2D and T2D in Both o-WAT and s-WAT

**Figure 3:**
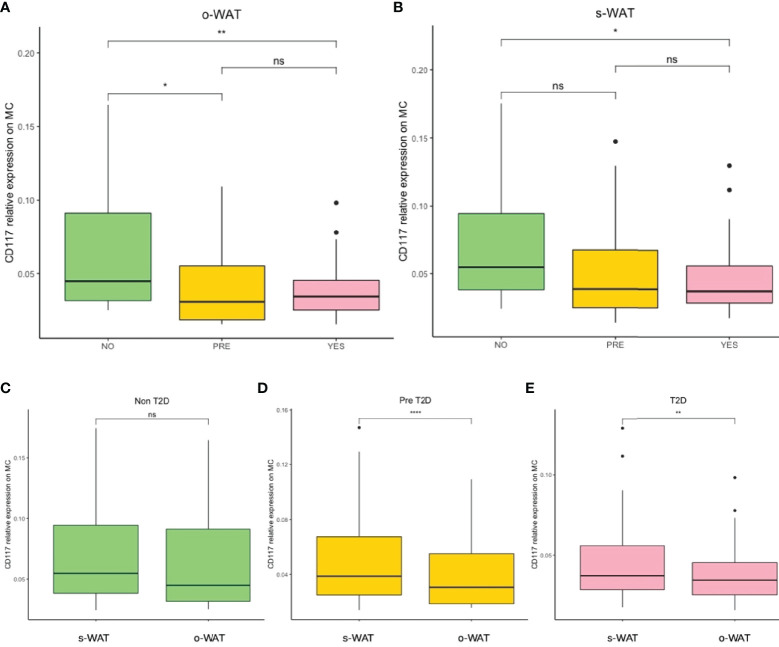
Relative surface expression of CD117 on mast cells. **(A)** Comparison in o-WAT between the three groups. **(B)** Comparison in s-WAT between the three groups. **(C)** Comparison between o-WAT and s-WAT in the non-T2D group. **(D)** Comparison between o-WAT and s-WAT in the pre-T2D group. **(E)** Comparison between o-WAT and s-WAT in the T2D group. MC (mast cells), s-WAT (subcutaneous white adipose tissue), o-WAT (omental white adipose tissue), NO (non-type 2 diabetes group), PRE (pre type 2 diabetes group), YES (type 2 diabetes group), T2D (type 2 diabetes), n=96. “ns” means not significant, * (0.05 > p value > 0.01), ** (0.01 > p value > 0.001), **** (0.0001 > p value > 0.00001).

**Figure 3** shows the surface expression of CD117 on mast cells in the three groups (n=96). In o-WAT (**Figure 3A**), there are statistically significant differences among non-T2D and T2D (p=0.0066) as well as between non-T2D and pre-T2D (p=0.036). Additionally, in s-WAT (**Figure 3B**), differences are only significant between non-T2D and T2D (p=0.046). Noteworthy, differences between o-WAT and s-WAT in the same patient (**Figures 3C–E**) were significant only in pre-T2D (p=4.114e-5) and T2D (p=0.0034). Nevertheless, in the case of non-T2D patients, differences were almost significant (0.071).

### The Surface Expression of FcϵRI on Mast Cells Decreases in Patients With T2D in o-WAT

**Figure 4:**
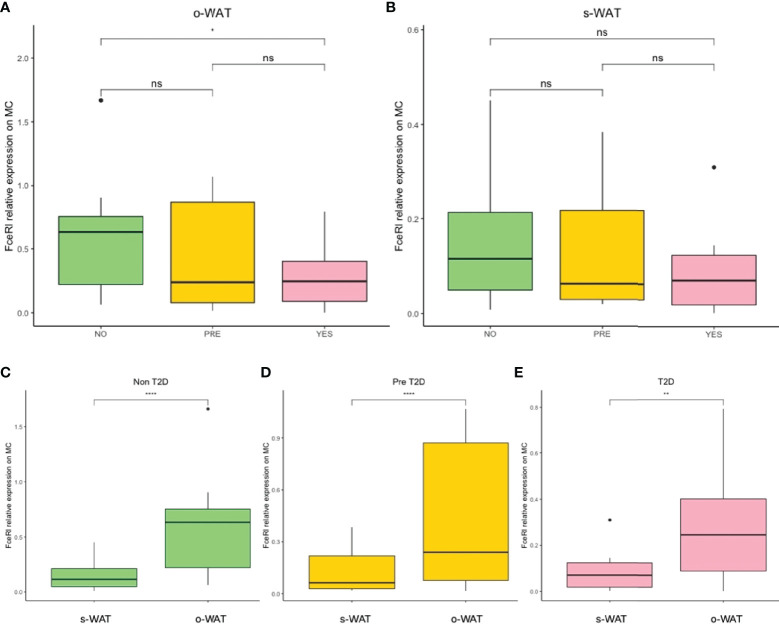
Relative surface expression of FceRI on mast cells. **(A)** Comparison in o-WAT between the three groups. **(B)** Comparison in s-WAT between the three groups. **(C)** Comparison between o-WAT and s-WAT in the non-T2D group. **(D)** Comparison between o-WAT and s-WAT in the pre-T2D group. **(E)** Comparison between o-WAT and s-WAT in the T2D group. MC (mast cells), s-WAT (subcutaneous white adipose tissue), o-WAT (omental white adipose tissue), NO (non-type 2 diabetes group), PRE (pre type 2 diabetes group), YES (type 2 diabetes group), T2D (type 2 diabetes), n=60. “ns” means not significant. * (0.05>p value>0.01), ** (0.01>p value>0.001), **** (0.0001>p value>0.00001).

**Figure 4** shows the surface expression of FcϵRI on mast cells in the three groups (n=60). In o-WAT (**Figure 4A**), there are statistically significant differences between non-T2D and T2D (p=0.043). Conversely, in s-WAT (**Figure 4B**), there are no significant differences among groups. However, in the comparison among o-WAT and s-WAT (**Figures 4C–E**), there are significant differences in non-T2D (p=4.289e-5), pre-T2D (p=8.367e-5), and T2D (p=0.0022). These results suggest that under normal conditions, mast cells have higher levels of FcϵRI in o-WAT than in s-WAT. However, T2D affects more the surface expression on mast cells in o-WAT than in s-WAT.

### The Surface Expression of CD203c on Mast Cells Decreases in Patients With T2D in o-WAT

**Figure 5:**
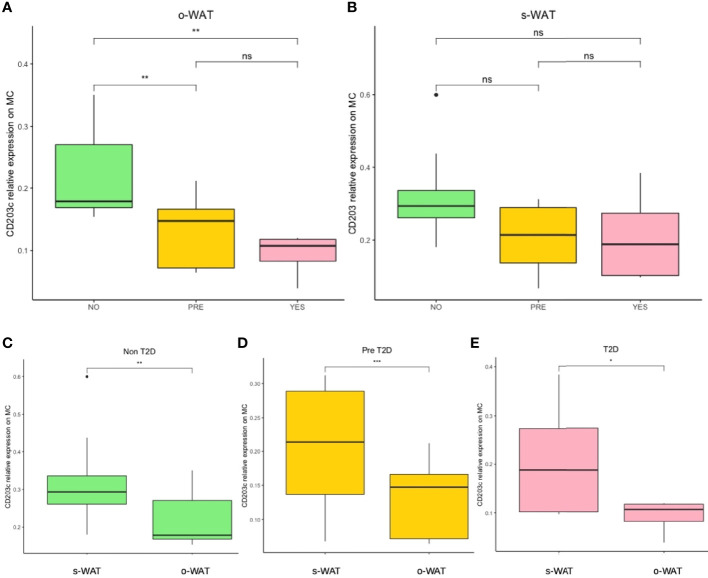
Relative surface expression of CD203c on mast cells. **(A)** Comparison in o-WAT between the three groups. **(B)** Comparison in s-WAT between the three groups. **(C)** Comparison between o-WAT and s-WAT in the non-T2D group. **(D)** Comparison between o-WAT and s-WAT in the pre-T2D group. **(E)** Comparison between o-WAT and s-WAT in the T2D group. MC (mast cells), s-WAT (subcutaneous white adipose tissue), o-WAT (omental white adipose tissue), NO (non-type 2 diabetes group), PRE (pre type 2 diabetes group), YES (type 2 diabetes group), T2D (type 2 diabetes), n=27. “ns” means not significant, * (0.05 > p value > 0.01), ** (0.01 > p value > 0.001), *** (0.001 > p value > 0.0001).

**Figure 5** shows the surface expression of CD203c on mast cells in the three groups (n=27). In o-WAT (**Figure 5A**), there are significant differences between non-T2D and T2D (p=0.0025), as well as between non-T2D and pre-T2D (p=0.0072). In s-WAT (**Figure 5B**), there are no significant differences between groups. Nevertheless, the latter may be due to the small sample size. Since the surface expression of CD203c in s-WAT is higher in non-T2D than in the other two groups, the chances are that increasing the cohort these differences will become statistically significant. These results indicate that the surface expression of CD203c on mast cells in o-WAT is highly affected by variations in glycemic control. The fact that so small p values were obtained with so little cohort indicates the strength of this phenomenon.

### The Gene Expression of CD117, FcϵRI, and CD203c in o-WAT and s-WAT Is Similar to Their Surface Expression

Since only mast cells express CD117, FcϵRI, and CD203c in adipose tissue, it was suitable to perform the RT-qPCR with RNA from the whole adipose tissue (n=60). However, since all leukocytes express CD45 in adipose tissue, it was not suitable to perform the RT-qPCR measurement.

**Figure 6:**
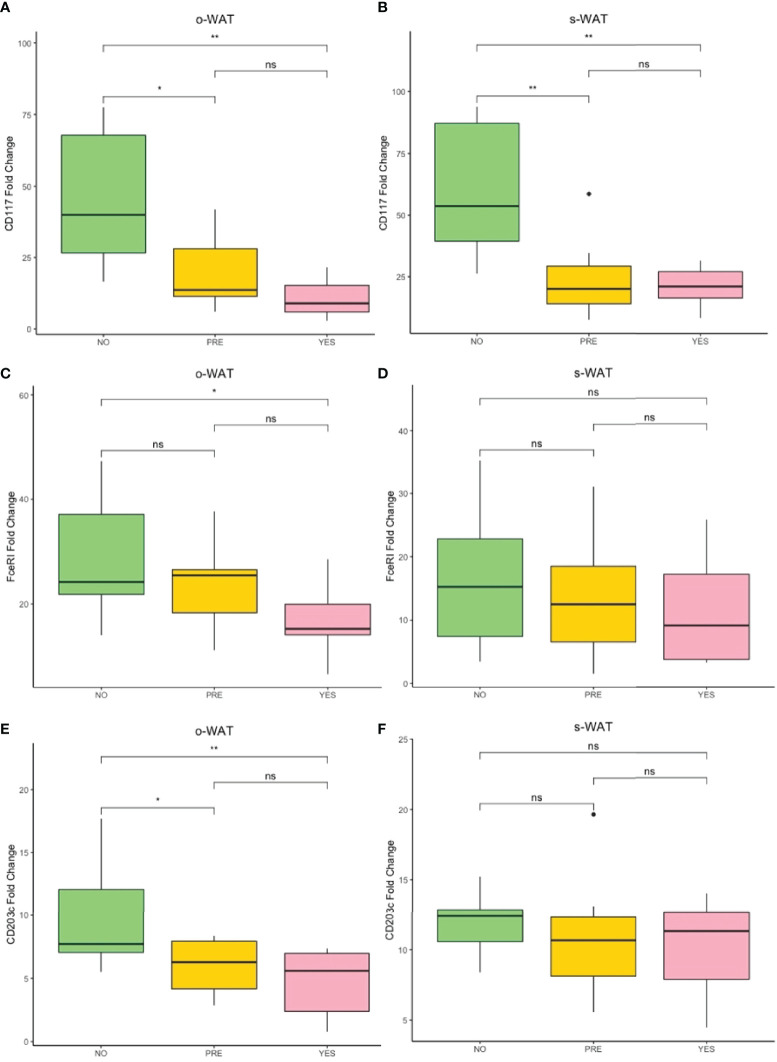
Fold change variation of the expression of CD117, FceRI, and CD203c. **(A)** Comparison of the fold change variation of CD117 in o-WAT between the three groups. **(B)** Comparison of the fold change variation of CD117 in s-WAT between the three groups. **(C)** Comparison of the fold change variation of FceRI in o-WAT between the three groups. **(D)** Comparison of the fold change variation of FceRI in s-WAT between the three groups. **(E)** Comparison of the fold change variation of CD203c in o-WAT between the three groups. **(F)** Comparison of the fold change variation of CD203c in s-WAT between the three groups. NO (non-type 2 diabetes group), PRE (pre type 2 diabetes group), YES (type 2 diabetes group), T2D (type 2 diabetes), s-WAT (subcutaneous white adipose tissue), o-WAT (omental white adipose tissue), n=60. “ns” means not significant, * (0.05 > p value > 0.01), ** (0.01 > p value > 0.001).

**Figure 6** shows the gene expression of CD117, FcϵRI, and CD203c. Noteworthy, the RT-qPCR determinations show the amount of mast cell mRNAs normalized with the expression level of tissue housekeeping genes. However, the cytometric analysis quantifies the surface density of several proteins of interest in a particular cytometric population. Although both methodologies target different steps of gene expression, the RT-qPCR data of CD117, FcϵRI, and CD203c follow the same trend as the surface expression analysis (**Figures 3**–**5**). Therefore, reinforcing the previous results about surface expression.

### The Gene Expression of β-hexosaminidase Decreases in Patients With T2D in o-WAT and s-WAT

**Figure 7:**
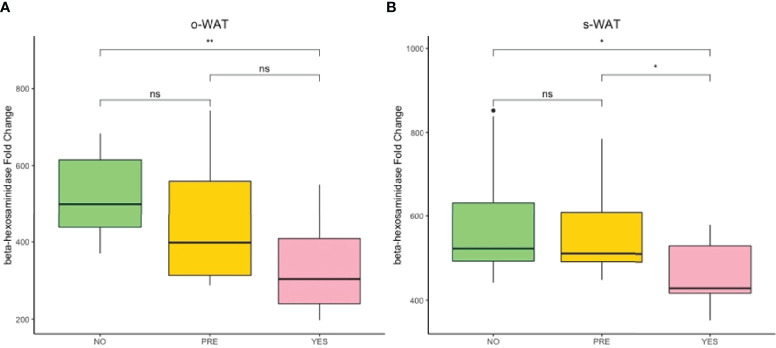
Fold change variation of the expression of β-hexosaminidase. **(A)** Comparison of the fold change variation of β-hexosaminidase in o-WAT between the three groups. **(B)** Comparison of the fold change variation of β-hexosaminidase in s-WAT between the three groups. NO (non-type 2 diabetes group), PRE (pre type 2 diabetes group), YES (type 2 diabetes group), T2D (type 2 diabetes), s-WAT (subcutaneous white adipose tissue), o-WAT (omental white adipose tissue), n=60. “ns” means not significant, * (0.05 > p value > 0.01), ** (0.01 > p value > 0.001).

**Figure 7** shows the gene expression of β-hexosaminidase in the three groups (n=60). In o-WAT (**Figure 7A**), there are significant differences between non-T2D and T2D (p=0.0043). In s-WAT, there are significant differences between non-T2D and T2D (p=0.023) as well as between pre-T2D and T2D (p=0.029). These results indicate that small changes in glycemic control have a huge impact on the expression of β-hexosaminidase, which is mainly produced by mast cells, in o-WAT and s-WAT.

### The Internal Structure of the Data Indicates That the Surface Expression of CD45 on Mast Cells in o-WAT Is a Good Predictor of T2D Status

**Figure 8:**
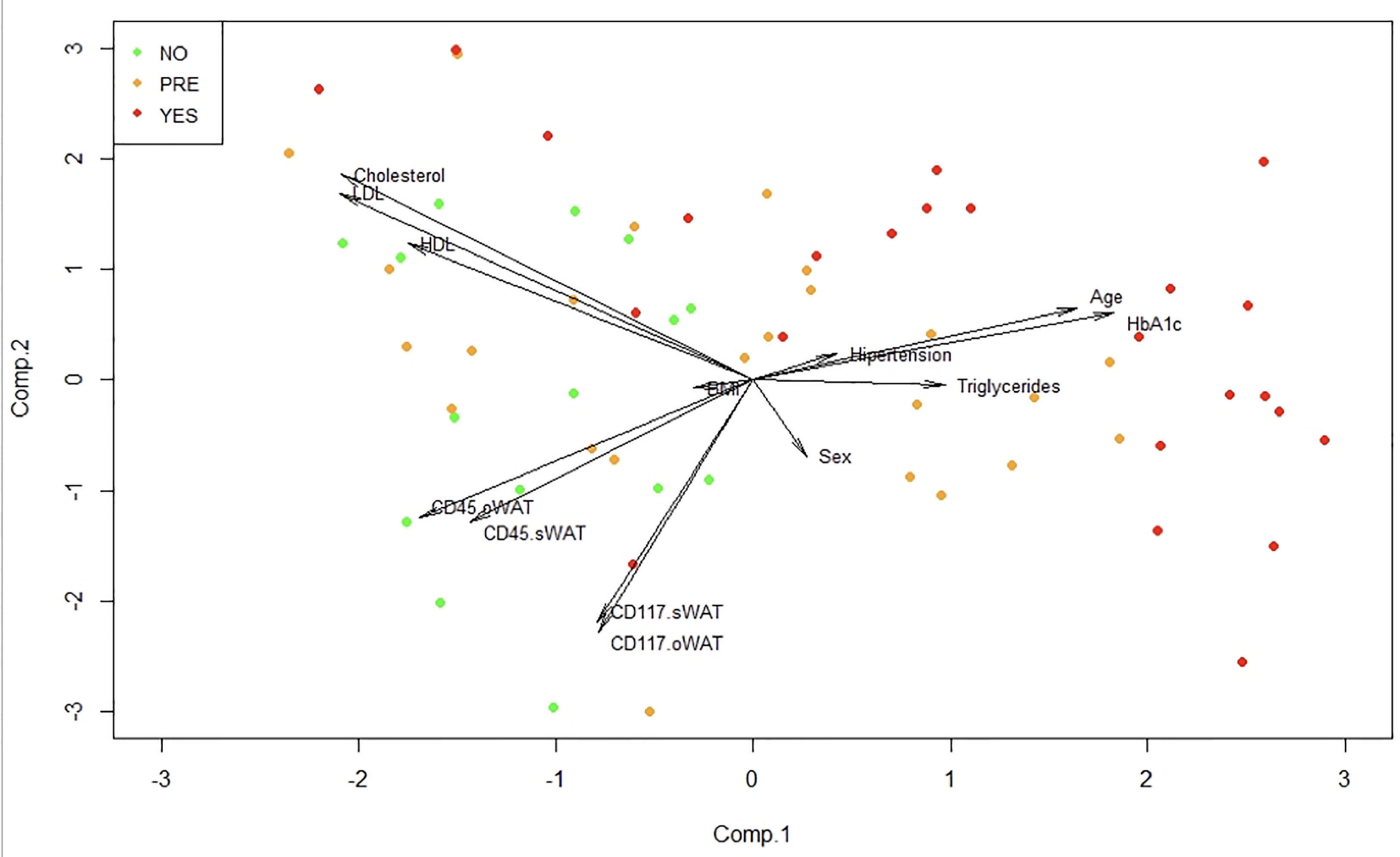
Principal component analysis (PCA). This technique allows the identification of patterns in our data by reducing its dimension. BMI (body mass index), oWAT (omental white adipose tissue), sWAT (subcutaneous white adipose tissue) Comp (component), NO (non-type 2 diabetes group), PRE (pre-type 2 diabetes group), YES (type 2 diabetes group), n=96.

To identify patterns in our high dimensional data set we employed a principal component analysis (PCA). In **Figure 8** it can be seen that patients of the non-T2D group occur in the left part of the plot of the two first principal components. After that, we performed a multiple regression analysis to observe if there is a relationship between the response variable HbA1c and any of the other variables of the study. Interestingly, we obtained significant p-values with age (p=0.0013) and CD45 on mast cells in o-WAT (p=0.031) in the individual t-test of significance. Additionally, the p-value of CD117 on mast cells in o-WAT was almost significant (p=0.066).

**Figure 9:**
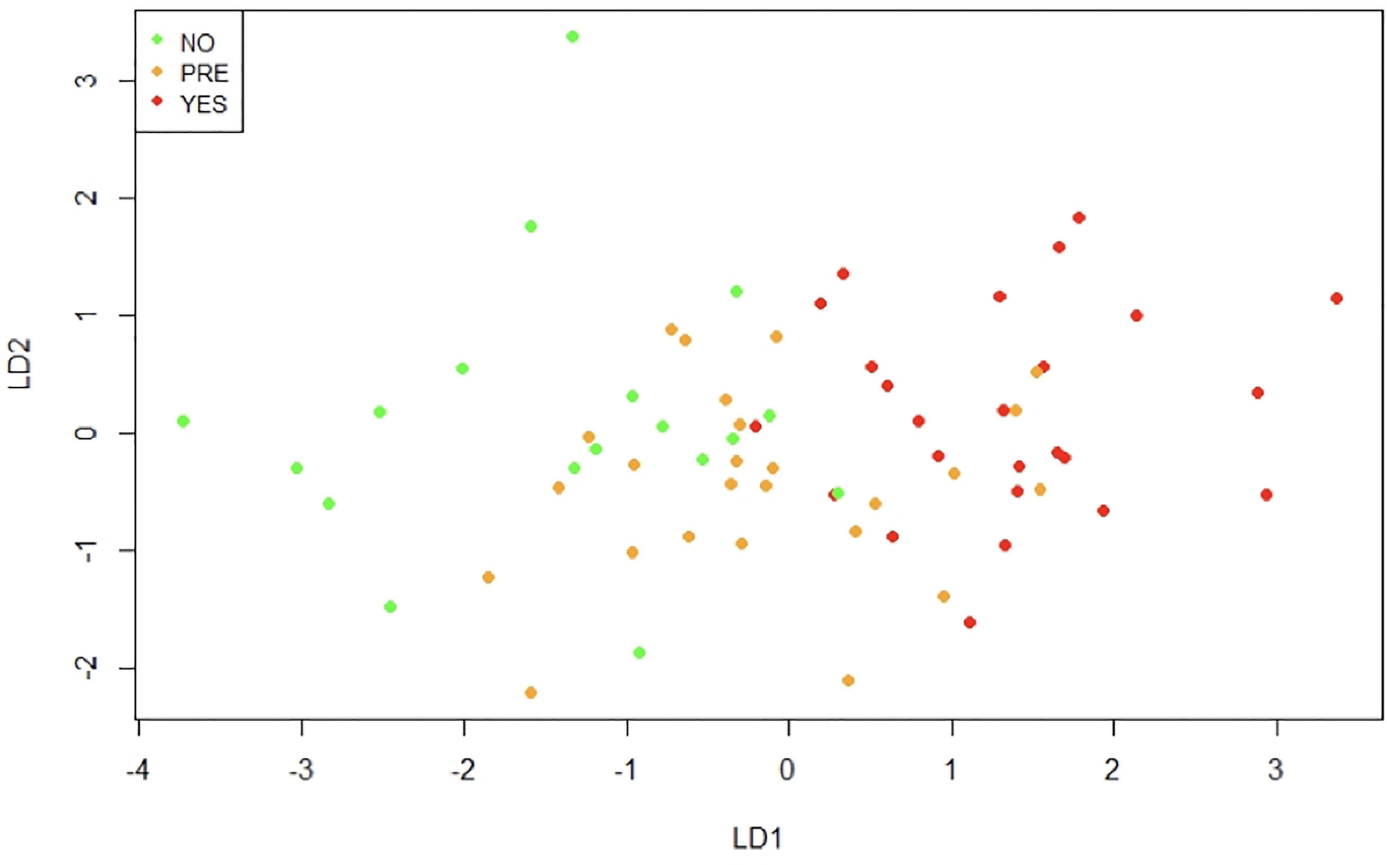
Linear discriminant analysis (LDA). This method identifies the linear combinations of variables that better separate the observations. NO (non-type 2 diabetes group), PRE (pre-type 2 diabetes group), YES (type 2 diabetes group), LD1 (linear discriminant 1), LD2 (linear discriminant 2), n=96.

Additionally, we performed a linear discriminant analysis. This allows finding the linear combinations of the original variables that can better separate the observations in the transformed space when considering the levels of “Type 2 Diabetes” variable (no, pre, yes). **Figure 9** shows that the first linear combination (LD1) separates reasonably well the observations. Patients in the non-T2D group have negative values for LD1, in the pre-T2D they have values around zero, and in the T2D they have positive values. The correlation of the variables with LD1 is displayed in **Table 2**. Noteworthy, the higher correlations are observed with CD45 on mast cells in o-WAT (-0.59), age (0.55), and CD117 on mast cells in o-WAT (-0.48).

**Table 2:** Correlation of each variable with LD1. BMI (body mass index), o-WAT (omental white adipose tissue), s-WAT (subcutaneous white adipose tissue), LD1 (linear discriminant 1).

Variable	Correlation with LD1
CD45 o-WAT	-0.588
Age	0.547
CD117 o-WAT	-0.485
CD117 s-WAT	-0.412
LDL	-0.393
CD45 s-WAT	-0.370
Cholesterol	-0.337
BMI	-0.294
Triglycerides	0.257
Hypertension	0.204
HDL	-0.125

**Figure 10:**
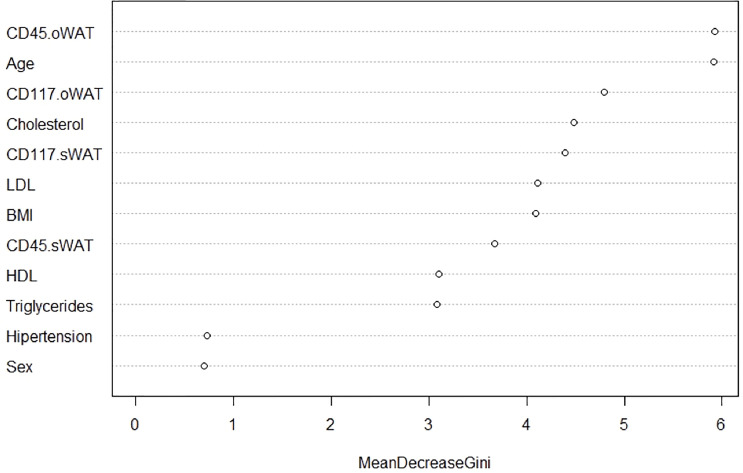
Random Forests Analysis. This method measures the relevance of the variables to discriminate the three levels of the categorical variable “Type 2 diabetes”. BMI (body mass index), o-WAT (omental white adipose tissue), s-WAT (subcutaneous white adipose tissue).

Finally, we employed a random forest analysis to identify the importance of each variable to discriminate between the three groups of patients. This importance is determined by the mean decrease in the Gini index. Compellingly, **Figure 10** shows that CD45 and CD117 on mast cells in o-WAT are respectively the first and third most important variables. Both of them are above the variables that measure lipid metabolism (cholesterol, triglycerides, HDL, and LDL) and BMI. These results highlight the strong relationship between mast cells in o-WAT and glycemic control.

## Discussion

Mast cells have intense post-transcriptional and post-translational regulation (18, 21). Within the post-translational regulation, they can sequester intracellularly membrane receptors (30–33). Besides, other proteins, like CD203c, are stored intracellularly and are translocated to the membrane only under certain circumstances (34). Therefore, traditional techniques like RT-PCR and Western Blot may overestimate the functional amount of some membrane proteins on mast cells. Here, we present a flow cytometry-based approach that allows us to simultaneously detect mast cells and their surface expression of CD45, CD117, CD203c, and FcϵRI. Since this technology only detects membrane-bound proteins, it can detect only the biologically active receptors. This method has two main advantages compared to traditional techniques like Western Blot. Firstly, it detects the above-mentioned proteins specifically on mast cells from the whole stromal vascular fraction. Secondly, it is not biased by cell number. Besides, since there is no purification step of mast cells, this approach is time and money-saving. This kind of technology has been employed before on mast cells (35), and it is also included in the diagnosis of some pathologies, including chronic granulomatous disease (36, 37). Additionally, usage and maintenance may cause oscillations in the lasers of the flow cytometer that may affect the accuracy of the results. Consequently, the internal standard removes this type of error and increases reproducibility.

The understanding of mast cells has evolved a lot in recent years. Mast cells are widely known for their role in allergy and inflammatory reactions (18). Thus, it was thought that they contribute to adipose tissue inflammation and T2D pathogenesis. To test this hypothesis, mast cell-free mice were developed by genetic ablation of CD117, the main protein for mast cell development and survival. Interestingly, mast cell-free mice had reduced obesity and glycemia. Accordingly, the number of mast cells was also associated with adipose tissue inflammation and T2D (25).

Later, different mast cell-free models were developed to corroborate the previous results. Importantly, it was observed that it was CD117 knockout and not mast cell absence that improved the metabolic profile in the previous model (26). Such occurs because CD117 is also important in the development of other cell types like lymphocytes (38, 39) and macrophages (40). Besides, other authors design other mast cell-free models and also observed that mast cells are not significant contributors to adipose tissue inflammation or insulin resistance (41, 42).

Noteworthy, the approaches of the models discussed before are focused on the immunological function of mast cells. However, mast cells play a crucial role in adipose tissue expansion. Since they promote both angiogenesis (17) and glucose-dependent adipogenesis (22, 23), they contribute to the metabolically healthy expansion of adipose tissue (adipocyte hyperplasia). Moreover, in the absence of mast cells, adipose tissue is poorly vascularized (43), which drives a metabolically unhealthy expansion *via* adipocyte hypertrophy (10, 11).

Once it was clear that mast cells are important players in metabolically healthy expansion and do not contribute to T2D we and others decided to investigate if T2D negatively affects mast cells. Previous studies of our group demonstrated that the number of mast cells decreases in patients with T2D, especially in o-WAT (27). Besides, we observed that after bariatric surgery, the number of mast cells increased 10-fold in o-WAT and 4-fold in s-WAT (44). Additionally, Goldstein et al. demonstrated that higher numbers of mast cells in adipose tissue are associated with higher weight loss after bariatric surgery (45). At this point, it was evident that T2D affects mast cells. Nonetheless, it was still unknown the specific phenotypic changes that T2D causes on mast cells.

Here, we demonstrate that mast cells also undergo phenotypic changes in patients with T2D. In agreement with previous results of the group (27, 44), in T2D, mast cells are more affected in o-WAT than in s-WAT. The decrease in the surface expression of CD117 in patients with T2D can explain the reduction in the number of mast cells. When the stem cell factor binds CD117 on the mast cell surface, it increases the expression of pro-survival proteins Bcl-2 and Bcl-XL while inactivate pro-apoptotic proteins Bad and Bim. Moreover, CD117 activation also enhances the activatory signals from other membrane receptors. When the surface expression of CD117 decreases, the survival and activatory signals for mast cells decrease too (46). Besides, low plasma levels of stem cell factor are associated with T2D and cardiometabolic diseases (47, 48). Therefore, mast cell death rate rises, and the number of mast cells decreases.

In the case of the decrease of FcϵRI on mast cells in patients with T2D, it can have two effects. Firstly, it decreases the possibility of mast cell activation *via* IgE-antigen binding in the surface of mast cells. To activate a mast cell, at least two IgE-FcϵRI complexes should be crosslinked (49). Therefore, the decrease in the surface expression of FcϵRI decreases the probability of this crosslinking event. Moreover, it has been observed that the binding of IgE to FcϵRI can promote mast cell survival even in the absence of stem cell factor (50, 51). Consequently, the decrease in the surface expression of FcϵRI can also contribute to the decrease in the number of mast cells in adipose tissue.

CD45 is a tyrosine phosphatase that critically regulates the activation status of Src family kinases (52). It has been observed that mutations in CD45 as well as changes in its expression can affect the effector function of mast cells (53). Previous studies demonstrated that CD45 deficient mast cells have an increase of inhibitory phosphorylations and also have dramatically impaired their effector functions (54). Besides, CD45 seems to play a role in proliferation. Interestingly, it is upregulated in mastocytosis (55). Since the surface expression of CD45 significantly decreases in o-WAT in pre-T2D and T2D groups, this indicates a decrease in the effector function of mast cells in o-WAT with subtle changes in the glycemic control. Consequently, mast cell activation is not an important player driving adipose tissue inflammation and malfunctioning in T2D, which agrees with previous reports (26, 41, 42). Besides, the decrease of the effector function of mast cells may affect their physiologic role in adipose tissue. This includes pre-adipocyte to adipocyte differentiation, lipid uptake by macrophages and their differentiation to foam cells, and angiogenesis.

One of the main functions of mast cells is their secretory activity, and this can be monitored with CD203c and β-hexosaminidase. CD203c is present only intracellularly on the membrane of granules (34). Therefore, CD203c only appears on the surface of mast cells after the release of granules. Unlike other markers, CD203c reaches the surface after every granule release and not only after classical degranulation (56). Regarding β-hexosaminidase, mast cells are the main producers in adipose tissue. Interestingly 85% is located in the granules and is frequently employed to measure mast cell degranulation *in vitro* (57). Therefore, the sharp decrease of CD203c and β-hexosaminidase observed in o-WAT indicates that mast cells have drastically reduced their secretory activity.

Taken together these results, in patients with T2D, there are phenotypic alterations of mast cells in adipose tissue (mainly in o-WAT). The decrease of FcϵRI, CD45, CD117, and CD203c on mast cells in o-WAT is associated with T2D and suggests a decline in their capacity to detect and respond to signals from the microenvironment. Such drastically compromises their homeostatic function in adipose tissue. Since the patients in the T2D group had a mild condition (HbA1c<7%), the decrease of the surface expression of FcϵRI, CD45, CD117, and CD203c may be sharper in patients with severe T2D. Nevertheless, since severe T2D is associated with a higher risk of complications during surgery, these patients frequently pursue other therapeutic approaches.

## Data Availability Statement

The raw data supporting the conclusions of this article will be made available by the authors, without undue reservation.

## Ethics Statement

The studies involving human participants were reviewed and approved by Andalucia’s Biomedical Research Ethics Committee of Granada (CEIM/CEI Granada). The patients/participants provided their written informed consent to participate in this study.

## Author Contributions

Data curation: DL-P and LG-E. Formal analysis: DL-P and LG-E. Funding acquisition: JL, JG, and ÁC. Investigation: DL-P, AR-R, CA, JS, JL, JG, and ÁC. Methodology: DL-P, AR-R, JG-R, and FT. Project administration: JL, JG, and ÁC. Supervision: JL, JG, and ÁC. Writing original draft: DL-P. Draft review and editing: DL-P, AR-R, JG-R, CA, LG-E, FT, JS, JL, JG, and ÁC. All authors contributed to the article and approved the submitted version.

## Funding

This work was supported by Instituto de Salud Carlos III (grants PI15/01361 and PI18/01947), and Conserjería de Salud y Familias, Junta de Andalucía (grant PIN-0235-2019). Instituto de Salud Carlos III (ISCIII) is a public organization that belongs to the Spanish Ministry of Health. ISCIII funds scientific projects on competitive open calls. Conserjería de Salud y Familias, Junta de Andalucía is a department of the regional government of Andalucía (Spain) that funds scientific projects on competitive open calls.

## Conflict of Interest

The authors declare that the research was conducted in the absence of any commercial or financial relationships that could be construed as a potential conflict of interest.

## Publisher’s Note

All claims expressed in this article are solely those of the authors and do not necessarily represent those of their affiliated organizations, or those of the publisher, the editors and the reviewers. Any product that may be evaluated in this article, or claim that may be made by its manufacturer, is not guaranteed or endorsed by the publisher.

## References

[1] Zheng Y Ley SH Hu FB. Global Aetiology and Epidemiology of Type 2 Diabetes Mellitus and its Complications. Nat Rev Endocrinol (2018) 14(2):88–98. doi: 10.1038/nrendo.2017.151 29219149

[2] Cho NH Shaw JE Karuranga S Huang Y da Rocha Fernandes JD Ohlrogge AW. IDF Diabetes Atlas: Global Estimates of Diabetes Prevalence for 2017 and Projections for 2045. Diabetes Res Clin Pract (2018) 138:271–81. doi: 10.1016/j.diabres.2018.02.023 29496507

[3] Harding JL Pavkov ME Magliano DJ Shaw JE Gregg EW. Global Trends in Diabetes Complications: A Review of Current Evidence. Diabetologia (2019) 62(1):3–16. doi: 10.1007/s00125-018-4711-2 30171279

[4] González-Salvatierra S García-Fontana C Andújar-Vera F Grau-Perales AB Martínez-Heredia L Avilés-Pérez MD. Osteoglycin as a Potential Biomarker of Mild Kidney Function Impairment in Type 2 Diabetes Patients. J Clin Med (2021) 10(10):1–10. doi: 10.3390/jcm10102209 PMC8161135 34065223

[5] Martin S. Caveolae, Lipid Droplets, and Adipose Tissue Biology: Pathophysiological Aspects. Hormone Mol Biol Clin Invest (2013) 15(1):11–8. doi: 10.1515/hmbci-2013-0035 25436728

[6] Jeffery E Wing A Holtrup B Sebo Z Kaplan JL Saavedra-Peña R. The Adipose Tissue Microenvironment Regulates Depot-Specific Adipogenesis in Obesity. Cell Metab (2016) 24(1):142–50. doi: 10.1016/j.cmet.2016.05.012 PMC4945385 27320063

[7] Donath MY Shoelson SE. Type 2 Diabetes as an Inflammatory Disease. Nat Rev Immunol (2011) 11(2):98–107. doi: 10.1038/nri2925 21233852

[8] Lackey DE Olefsky JM. Regulation of Metabolism by the Innate Immune System. Nat Rev Endocrinol (2016) 12(1):15–28. doi: 10.1038/nrendo.2015.189 26553134

[9] Lee JJ Beretvas SN Freeland-Graves JH. Abdominal Adiposity Distribution in Diabetic/Prediabetic and Nondiabetic Populations: A Meta-Analysis. J Obes (2014) 2014:1–20. doi: 10.1155/2014/697264 PMC4261846 25525511

[10] Choe SS Huh JY Hwang IJ Kim JI Kim JB. Adipose Tissue Remodeling: Its Role in Energy Metabolism and Metabolic Disorders. Front Endocrinol (2016) 7:3389/fendo.2016.00030. doi: 10.3389/fendo.2016.00030 PMC4829583 27148161

[11] Kusminski CM Bickel PE Scherer PE. Targeting Adipose Tissue in the Treatment of Obesity-Associated Diabetes. Nat Rev Drug Discovery (2016) 15(9):639–60. doi: 10.1038/nrd.2016.75 27256476

[12] Guerrini V Gennaro ML. Foam Cells: One Size Doesn’t Fit All. Trends Immunol (2019) 40(12):1163–79. doi: 10.1016/j.it.2019.10.002 PMC6925453 31732284

[13] Prieur X Mok CYL Velagapudi VR Núñez V Fuentes L Montaner D. Differential Lipid Partitioning Between Adipocytes and Tissue Macrophages Modulates Macrophage Lipotoxicity and M2/M1 Polarization in Obese Mice. Diabetes (2011) 60(3):797–809. doi: 10.2337/db10-0705 21266330 PMC3046840

[14] Li Y Wong K Giles A Jiang J Lee JW Adams AC. Hepatic SIRT1 Attenuates Hepatic Steatosis and Controls Energy Balance in Mice by Inducing Fibroblast Growth Factor 21. Gastroenterology (2014) 146(2):539–49. doi: 10.1053/j.gastro.2013.10.059 PMC4228483 24184811

[15] Perry RJ Camporez J-PG Kursawe R Titchenell PM Zhang D Perry CJ. Hepatic Acetyl CoA Links Adipose Tissue Inflammation to Hepatic Insulin Resistance and Type 2 Diabetes. Cell (2015) 160(4):745–58. doi: 10.1016/j.cell.2015.01.012 PMC4498261 25662011

[16] Cheng R Ma J. Angiogenesis in Diabetes and Obesity. Rev Endocrine Metab Disord (2015) 16(1):67–75. doi: 10.1007/s11154-015-9310-7 25663658 PMC4351724

[17] Krystel-Whittemore M Dileepan KN Wood JG. Mast Cell: A Multi-Functional Master Cell. Front Immunol (2016) 6:3389/fimmu.2015.00620. doi: 10.3389/fimmu.2015.00620 PMC4701915 26779180

[18] Gilfillan AM Beaven MA. Regulation of Mast Cell Responses in Health and Disease. Crit Rev Immunol (2011) 31(6):475–530. doi: 10.1615/critrevimmunol.v31.i6.30 22321108 PMC3395887

[19] Mukai K Tsai M Saito H Galli SJ. Mast Cells as Sources of Cytokines, Chemokines, and Growth Factors. Immunol Rev (2018) 282(1):121–50. doi: 10.1111/imr.12634 PMC5813811 29431212

[20] Wernersson S Pejler G. Mast Cell Secretory Granules: Armed for Battle. Nat Rev Immunol (2014) 14(7):478–94. doi: 10.1038/nri3690 24903914

[21] Rönnberg E Melo FR Pejler G. Mast Cell Proteoglycans. J Histochem Cytochem (2012) 60(12):950–62. doi: 10.1369/0022155412458927 PMC3527880 22899859

[22] Tanaka A Nomura Y Matsuda A Ohmori K Matsuda H. Mast Cells Function as an Alternative Modulator of Adipogenesis Through 15-Deoxy-Delta-12, 14-Prostaglandin J 2. Am J Physiology-Cell Physiol (2011) 301(6):1360–7. doi: 10.1152/ajpcell.00514.2010 21865589

[23] Shi MA Shi G-P. Different Roles of Mast Cells in Obesity and Diabetes: Lessons From Experimental Animals and Humans. Front Immunol (2012) 3:3389/fimmu.2012.00007. doi: 10.3389/fimmu.2012.00007 PMC3341969 22566893

[24] Jung U Choi M-S. Obesity and Its Metabolic Complications: The Role of Adipokines and the Relationship Between Obesity, Inflammation, Insulin Resistance, Dyslipidemia and Nonalcoholic Fatty Liver Disease. Int J Mol Sci (2014) 15(4):6184–223. doi: 10.3390/ijms15046184 PMC4013623 24733068

[25] Liu J Divoux A Sun J Zhang J Clément K Glickman JN. Genetic Deficiency and Pharmacological Stabilization of Mast Cells Reduce Diet-Induced Obesity and Diabetes in Mice. Nat Med (2009) 15(8):940–5. doi: 10.1038/nm.1994 PMC2736875 19633655

[26] Gutierrez DA Muralidhar S Feyerabend TB Herzig S Rodewald H-R. Hematopoietic Kit Deficiency, Rather Than Lack of Mast Cells, Protects Mice From Obesity and Insulin Resistance. Cell Metab (2015) 21(5):678–91. doi: 10.1016/j.cmet.2015.04.013 25955205

[27] Lopez-Perez D Redruello-Romero A Garcia-Rubio J Arana C Garcia-Escudero LA Tamayo F. In Patients With Obesity, the Number of Adipose Tissue Mast Cells Is Significantly Lower in Subjects With Type 2 Diabetes. Front Immunol (2021) 12:3389/fimmu.2021.664576. doi: 10.3389/fimmu.2021.664576 PMC8177010 34093556

[28] American Diabetes Association. 2. Classification and Diagnosis of Diabetes: Standards of Medical Care in Diabetes. Diabetes Care (2019) 42(Supplement 1):13–28. doi: 10.2337/dc19-S002 30559228

[29] American Diabetes Association. Standards of Medical Care in Diabetes. Diabetes Care (2014) 37(Supplement_1):14–80. doi: 10.2337/dc14-S014

[30] Kornstädt L Pierre S Weigert A Ebersberger S Schäufele TJ Kolbinger A. Bacterial and Fungal Toll-Like Receptor Activation Elicits Type I IFN Responses in Mast Cells. Front Immunol (2021) 11:3389/fimmu.2020.607048. doi: 10.3389/fimmu.2020.607048 PMC7907501 33643293

[31] Migalovich-Sheikhet H Friedman S Mankuta D Levi-Schaffer F. Novel Identified Receptors on Mast Cells. Front Immunol (2012) 3:3389/fimmu.2012.00238. doi: 10.3389/fimmu.2012.00238 PMC3410575 22876248

[32] Murakami T Suzuki K Niyonsaba F Tada H Reich J Tamura H. MrgX2-mediated Internalization of LL-37 and Degranulation of Human LAD2 Mast Cells. Mol Med Rep (2018) 18:4951–9. doi: 10.3892/mmr.2018.9532 PMC6236315 30280189

[33] Bulfone-Paus S Nilsson G Draber P Blank U Levi-Schaffer F. Positive and Negative Signals in Mast Cell Activation. Trends Immunol (2017) 38(9):657–67. doi: 10.1016/j.it.2017.01.008 28254170

[34] Valent P Cerny-Reiterer S Herrmann H Mirkina I George TI Sotlar K. Phenotypic Heterogeneity, Novel Diagnostic Markers, and Target Expression Profiles in Normal and Neoplastic Human Mast Cells. Best Pract Res Clin Haematology (2010) 23(3). doi: 10.1016/j.beha.2010.07.003 21112036

[35] Cop N Decuyper I Faber M Sabato V Bridts C Hagendorens M. Phenotypic and Functional Characterization of *In Vitro* Cultured Human Mast Cells. Cytometry Part B: Clin Cytometry (2017) 92(5). doi: 10.1002/cyto.b.21399 27401129

[36] O’Gorman MR Corrochano V. Rapid Whole-Blood Flow Cytometry Assay for Diagnosis of Chronic Granulomatous Disease. Clin Diagn Lab Immunol (1995) 2(2). doi: 10.1128/cdli.2.2.227-232.1995 PMC170133 7697534

[37] Baris HE Ogulur I Akcam B Kiykim A Karagoz D Saraymen B. Diagnostic Modalities Based on Flow Cytometry for Chronic Granulomatous Disease: A Multicenter Study in a Well-Defined Cohort. J Allergy Clin Immunology: In Pract (2020) 8(10). doi: 10.1016/j.jaip.2020.07.030 32736065

[38] Massa S Balciunaite G Ceredig R Rolink AG. Critical Role for C-Kit (CD117) in T Cell Lineage Commitment and Early Thymocyte Development*In Vitro* . Eur J Immunol (2006) 36(3):526–32. doi: 10.1002/eji.200535760 16482516

[39] Frumento G Zuo J Verma K Croft W Ramagiri P Chen FE. CD117 (C-Kit) Is Expressed During CD8+ T Cell Priming and Stratifies Sensitivity to Apoptosis According to Strength of TCR Engagement. Front Immunol (2019) 10:3389/fimmu.2019.00468. doi: 10.3389/fimmu.2019.00468 PMC6428734 30930902

[40] Lambert C Preijers FWMB Yanikkaya Demirel G Sack U. Monocytes and Macrophages in Flow: An ESCCA Initiative on Advanced Analyses of Monocyte Lineage Using Flow Cytometry. Cytometry Part B: Clin Cytometry (2017) 92(3):180–8. doi: 10.1002/cyto.b.21280 26332381

[41] Chmelař J Chatzigeorgiou A Chung K-J Prucnal M Voehringer D Roers A. No Role for Mast Cells in Obesity-Related Metabolic Dysregulation. Front Immunol (2016) 7:3389/fimmu.2016.00524. doi: 10.3389/fimmu.2016.00524 PMC5121122 27933062

[42] Elieh Ali Komi D Shafaghat F Christian M. Crosstalk Between Mast Cells and Adipocytes in Physiologic and Pathologic Conditions. Clin Rev Allergy Immunol (2020) 58(3):388–400. doi: 10.1007/s12016-020-08785-7 32215785 PMC7244609

[43] Mraz M Haluzik M. The Role of Adipose Tissue Immune Cells in Obesity and Low-Grade Inflammation. J Endocrinol (2014) 222(3):113–27. doi: 10.1530/JOE-14-0283 25006217

[44] García-Rubio J León J Redruello-Romero A Pavón E Cozar A Tamayo F. Cytometric Analysis of Adipose Tissue Reveals Increments of Adipocyte Progenitor Cells After Weight Loss Induced by Bariatric Surgery. Sci Rep (2018) 8(1):1–10. doi: 10.1038/s41598-018-33488-7 30315279 PMC6185966

[45] Goldstein N Kezerle Y Gepner Y Haim Y Pecht T Gazit R. Higher Mast Cell Accumulation in Human Adipose Tissues Defines Clinically Favorable Obesity Sub-Phenotypes. Cells (2020) 9(6):1–12. doi: 10.3390/cells9061508 PMC7349306 32575785

[46] Caslin HL Kiwanuka KN Haque TT Taruselli MT MacKnight HP Paranjape A. Controlling Mast Cell Activation and Homeostasis: Work Influenced by Bill Paul That Continues Today. Front Immunol (2018) 9:3389/fimmu.2018.00868. doi: 10.3389/fimmu.2018.00868 PMC5932183 29755466

[47] Björkbacka H Yao Mattisson I Wigren M Melander O Fredrikson GN Bengtsson E. Plasma Stem Cell Factor Levels are Associated With Risk of Cardiovascular Disease and Death. J Internal Med (2017) 282(6):508–21. doi: 10.1111/joim.12675 28842933

[48] Bouzina H Rådegran G. Low Plasma Stem Cell Factor Combined With High Transforming Growth Factor-α Identifies High-Risk Patients in Pulmonary Arterial Hypertension. ERJ Open Res (2018) 4(4):1–9. doi: 10.1183/23120541.00035-2018 PMC6230818 30443557

[49] Sibilano R Frossi B Pucillo CE. Mast Cell Activation: A Complex Interplay of Positive and Negative Signaling Pathways. Eur J Immunol (2014) 44(9):2558–66. doi: 10.1002/eji.201444546 25066089

[50] Asai K Kitaura J Kawakami Y Yamagata N Tsai M Carbone DP. Regulation of Mast Cell Survival by IgE. Immunity (2001) 14(6):791–800. doi: 10.1016/S1074-7613(01)00157-1 11420048

[51] Bax HJ Keeble AH Gould HJ. Cytokinergic IgE Action in Mast Cell Activation. Front Immunol (2012) 3:3389/fimmu.2012.00229. doi: 10.3389/fimmu.2012.00229 PMC3412263 22888332

[52] Rheinländer A Schraven B Bommhardt U. CD45 in Human Physiology and Clinical Medicine. Immunol Lett (2018) 196:22–32. doi: 10.1016/j.imlet.2018.01.009 29366662

[53] Berger SA Mak TW Paige CJ. Leukocyte Common Antigen (CD45) is Required for Immunoglobulin E-Mediated Degranulation of Mast Cells. J Exp Med (1994) 180(2):471–6. doi: 10.1084/jem.180.2.471 PMC2191589 8046327

[54] Grochowy G Hermiston ML Kuhny M Weiss A Huber M. Requirement for CD45 in Fine-Tuning Mast Cell Responses Mediated by Different Ligand–Receptor Systems. Cell Signalling (2009) 21(8):1277–86. doi: 10.1016/j.cellsig.2009.03.018 19332117

[55] Chisholm KM Merker JD Gotlib JR Gitana G Lefterova M Zehnder JL. Mast Cells in Systemic Mastocytosis Have Distinctly Brighter CD45 Expression by Flow Cytometry. Am J Clin Pathol (2015) 143(4):527–34. doi: 10.1309/AJCPZ3J4GEEYIRRA 25780004

[56] Metcalfe DD Pawankar R Ackerman SJ Akin C Clayton F Falcone FH. Biomarkers of the Involvement of Mast Cells, Basophils and Eosinophils in Asthma and Allergic Diseases. World Allergy Organ J (2016) 9:1–5. doi: 10.1186/s40413-016-0094-3 26904159 PMC4751725

[57] Fukuishi N Murakami S Ohno A Yamanaka N Matsui N Fukutsuji K. Does β-Hexosaminidase Function Only as a Degranulation Indicator in Mast Cells? The Primary Role of β-Hexosaminidase in Mast Cell Granules. J Immunol (2014) 193(4):1886–94. doi: 10.4049/jimmunol.1302520 25015817

