# Supplementary material for: In Obese Patients With Type 2 Diabetes, Mast Cells in Omental Adipose Tissue Decrease the Surface Expression of CD45, CD117, CD203c, and FcϵRI
Source: Front Endocrinol (Lausanne). 2022 Mar 15;13:818388. doi: 10.3389/fendo.2022.818388 (PMC8965342; doi:10.3389/fendo.2022.818388)
Supplement: Supplementary file 1 [file DataSheet_1.docx]

Supplementary Material

# Supplementary Figures and Tables


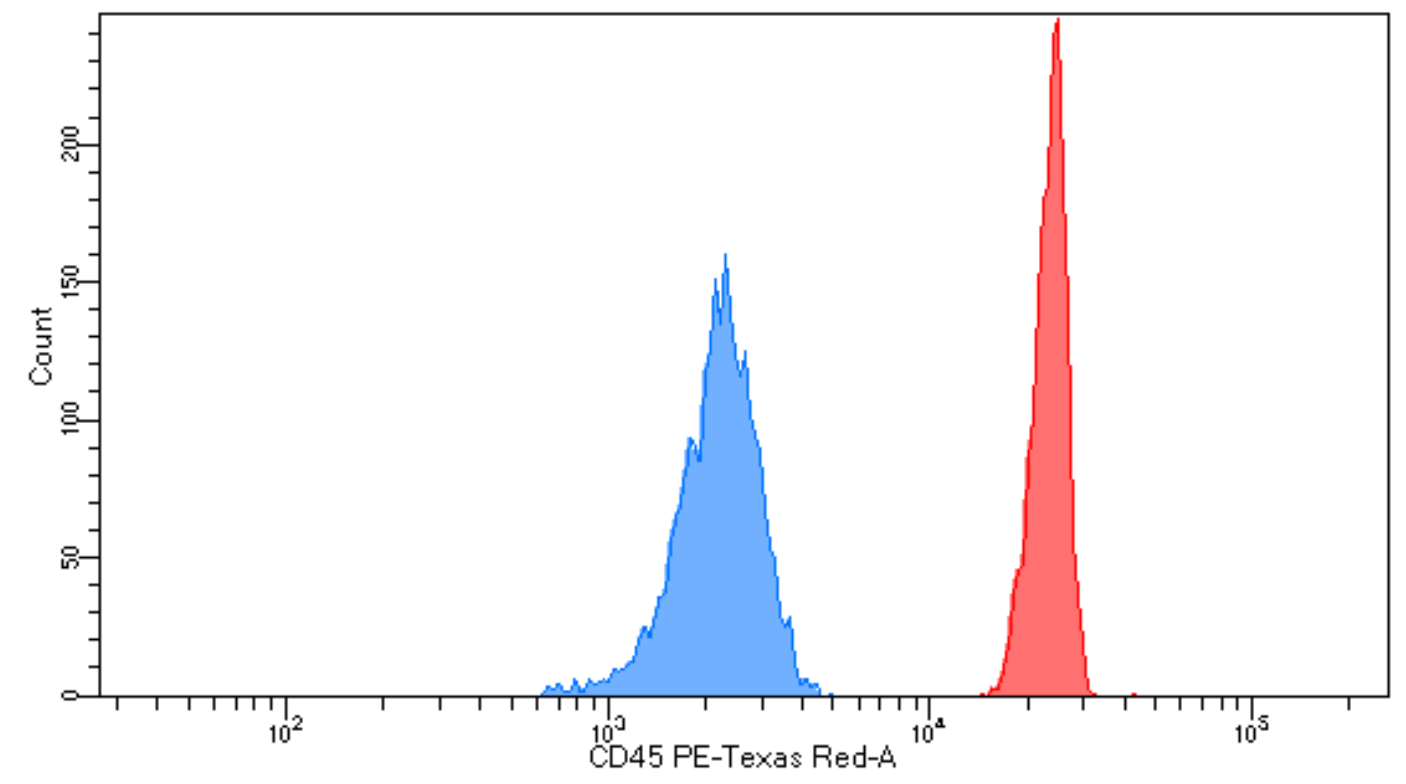


**Supplementary Figure 1.** Histogram of the fluorescence signal of the internal standard (red), and CD45 on the surface of mast cells (blue).


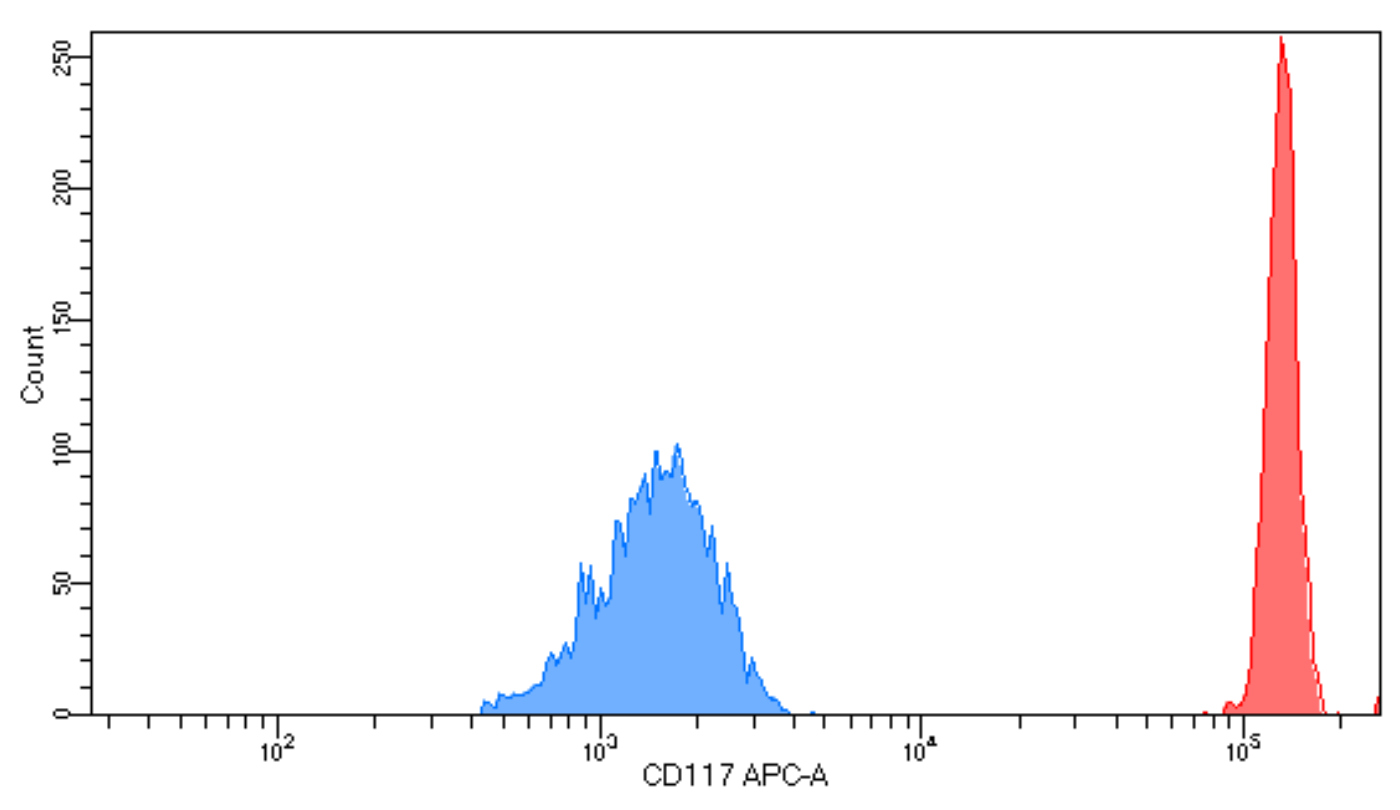


**Supplementary Figure 2**. Histogram of the fluorescence signal of the internal standard (red), and CD117 on the surface of mast cells (blue).


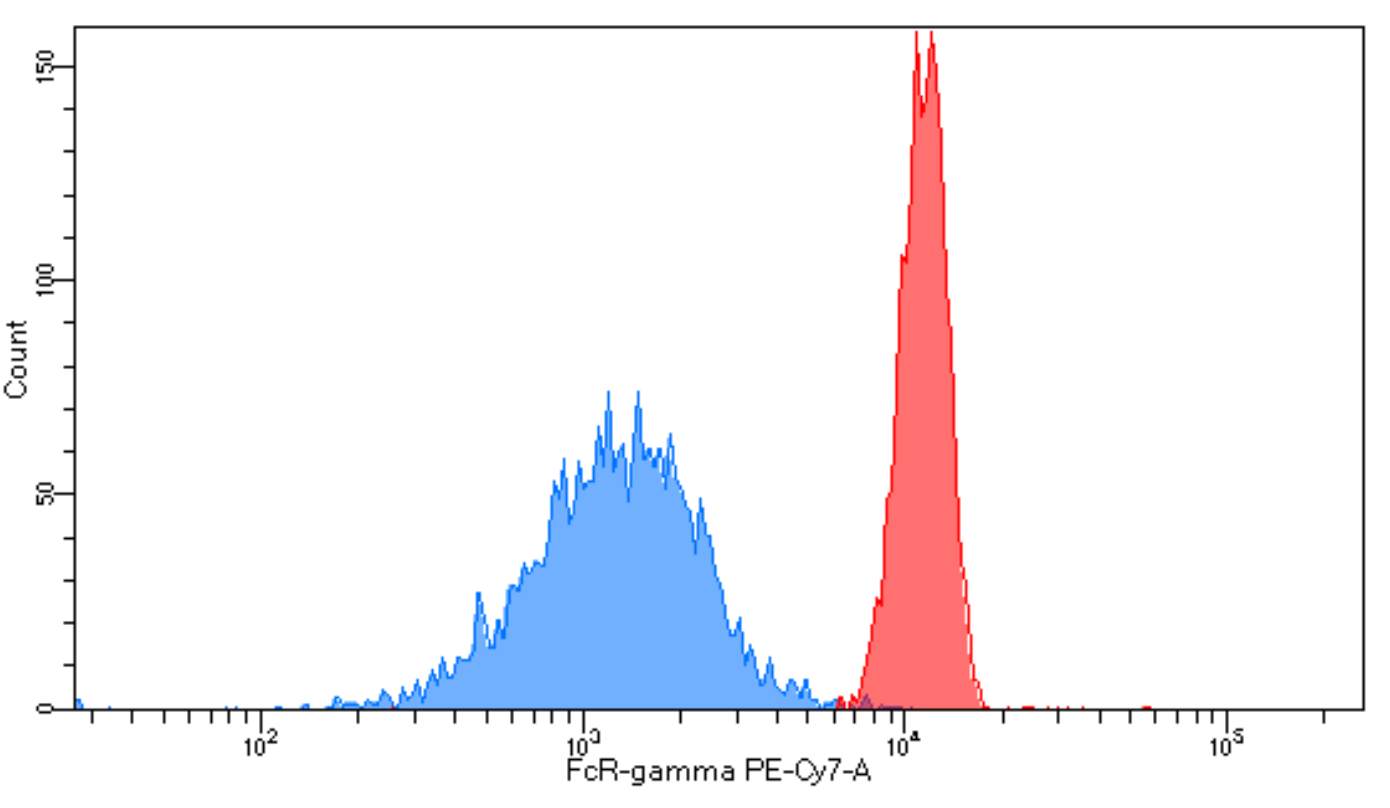
**Supplementary Figure 3**. Histogram of the fluorescence signal of the internal standard (red), and FceRI on the surface of mast cells (blue).


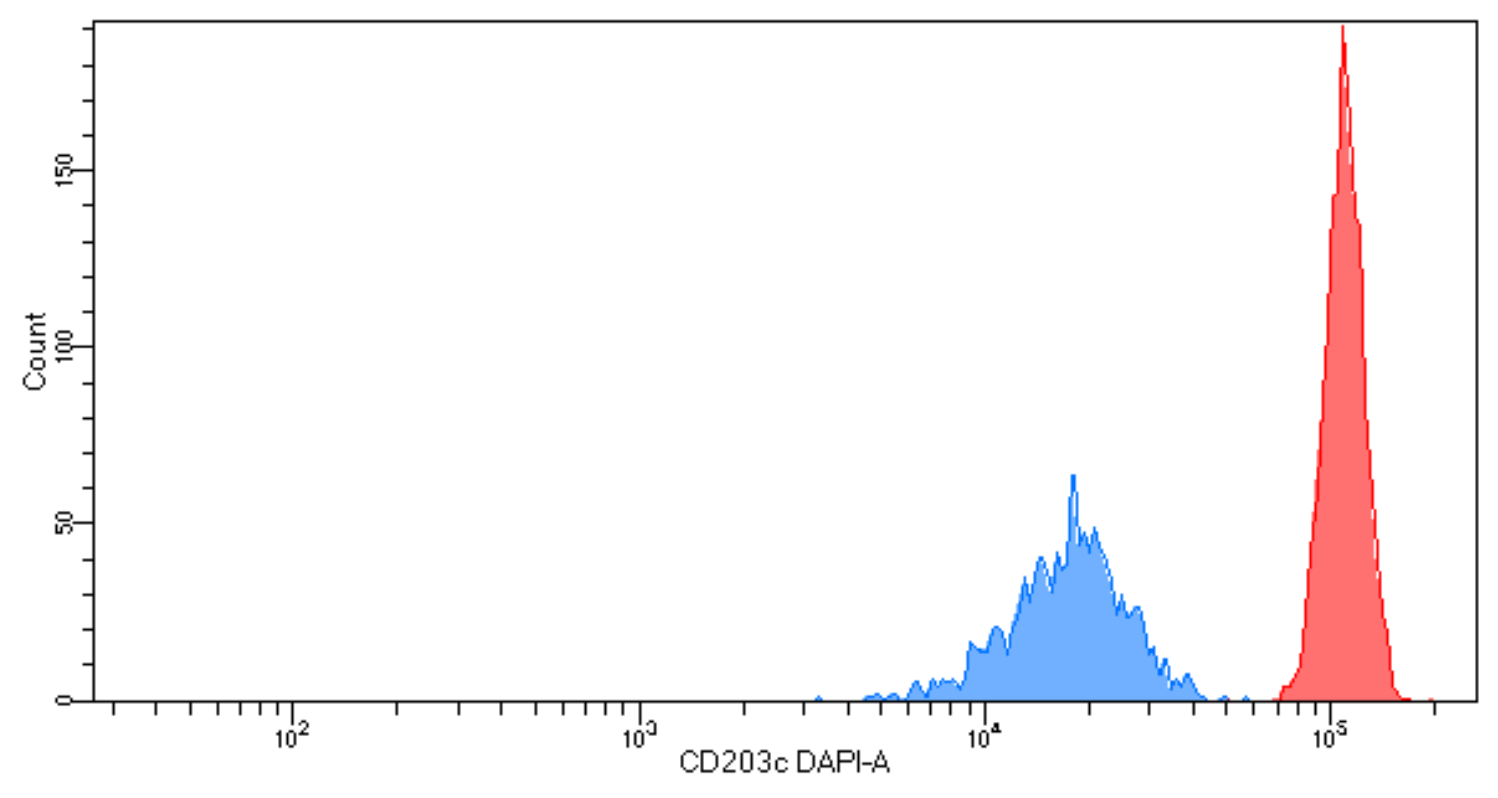


**Supplementary Figure 4**. Histogram of the fluorescence signal of the internal standard (red), and CD203c on the surface of mast cells (blue).

**Supplementary Table 1**. Sequences of the primers employed in the qPCR

| **Primer** | **Sequence** |
| --- | --- |
| Sen-CD117 | gcagatttcagagagcaccaatcatattta |
| Ant-CD117 | aagagatcattcctggaggggtgacccaa |
| Sen CD203c | gatgctccagatgaaattaccaaacattta |
| Ant CD203c | ccaaagagcttctggtttaccttcaggac |
| Sen FceRI | cgctccagatggcgtgttagcagtccc |
| Ant FceRI | ggtgctgacatttgtattctccactgtctt |
| Sen HEX-B | agtggaacctcttgattttggcggtactc |
| Ant HEX-B | ggttgtgcagctattccacgttcgaccat |
| Sen PPIA | ccatggcaaatgctggacccaacacaaatg |
| Ant PPIA | tcctgagctacagaaggaatgatctggtgg |
| Sen RPS13 | ggtgttgcacaagtacgttttgtgacaggc |
| Ant RPS13 | tcatatttccaattgggagggaggactcgc |
